# Effect of GP19 Peptide Hyperimmune Antiserum on Activated Macrophage during *Ehrlichia canis* Infection in Canine Macrophage-like Cells

**DOI:** 10.3390/ani11082310

**Published:** 2021-08-05

**Authors:** Boondarika Nambooppha, Amarin Rittipornlertrak, Anucha Muenthaisong, Pongpisid Koonyosying, Sahatchai Tangtrongsup, Saruda Tiwananthagorn, Yang-Tsung Chung, Nattawooti Sthitmatee

**Affiliations:** 1 Department of Veterinary Biosciences and Veterinary Public Health, Faculty of Veterinary Medicine, Chiang Mai University, Chiang Mai 50100, Thailand; boondarika.n@gmail.com (B.N.); mate_vet@hotmail.com (A.R.); anucharham@gmail.com (A.M.); pk404mc@hotmail.com (P.K.); stiwananthagorn@gmail.com (S.T.); 2 Department of Companion Animals and Wildlife Clinics, Faculty of Veterinary Medicine, Chiang Mai University, Chiang Mai 50100, Thailand; sahatchai.t@cmu.ac.th; 3 Department of Veterinary Medicine, College of Veterinary Medicine, National Chung Hsing University, Taichung 402, Taiwan; rjchung_097@yahoo.com.tw; 4 Excellence Center in Veterinary Bioscience, Chiang Mai University, Chiang Mai 50100, Thailand

**Keywords:** *Ehrlichia canis*, cytokine, DH82, GP19, hyperimmune, macrophage-like cells, peptide

## Abstract

**Simple Summary:**

The commercial canine ehrlichiosis vaccine is still not available. A limitation of developing vaccines is the knowledge of the humoral immune response against *E. canis* infection is still unknown. In this study, we designed the peptide vaccine candidate, namely GP19_4-43_, for inducing hyperimmune serum in rabbits. The rabbit sera were used to examine for *E. canis* infective inhibition in macrophage-like cells (DH82). A decrease in *E. canis* infection (<50% of infection) was observed on DH82 cells in the treated group with GP19_4-43_ antiserum on the third day of the post-infection period. Cytokine genes in DH82 with/without rabbit sera were investigated and showed marked up-regulation of the *IFNG* expression level in DH82 cells in the treated group with GP19_4-43_ antiserum. This study provides the preliminary information of immune response against *E. canis* of immunized animals and directions for genomic or/and proteomic studies involved in phagocyte-cell mediated immune response.

**Abstract:**

In terms of its veterinary importance, vaccine development against *Ehrlichia canis* is needed. However, the effect of developing vaccines on humoral immune response against *E. canis* infection is still unknown. Novel GP19_4-43_ was synthesized according to *E. canis* GP19 epitope prediction. To restrict any loss and/or illness in the host animal, rabbits were used in this study to produce GP19_4-43_ hyperimmune sera. The effect of GP19_4-43_ hyperimmune sera on neutralization was examined in vitro by determining the inhibition of *E. canis* infection of the macrophage-like cell line (DH82) in the presence of the sera. Four groups of DH82 cells received differing treatments. These included *E. canis* experimentally infected DH82 cells, *E. canis*-infected DH82 cells with control rabbit serum (untreated group), *E. canis*-infected DH82 cells with GP19_4-43_ rabbit antiserum (treated group) and uninfected cells (negative control group), respectively. The treated group developed a decrease (*p* < 0.01) in the percentage of *E. canis* infected cells after 3 days post-infection at 48.57 ± 1.28. In addition, real-time PCR analyses of cytokine mRNA expression involved with the macrophage, humoral, and cellular immune responses were conducted. The findings revealed an upregulated expression of *IFNG* in the treated group during the infection. This study demonstrated neutralization in the GP19_4-43_ peptide hyperimmune sera of immunized rabbits. Notably, IFN-γ production could be effectively promoted in canine macrophages in relation to the activation of macrophages and adaptive immune responses. The results of this study indicate the potential for the use of this immunogen in further investigations involving immunized and infected dogs as *E. canis* host species.

## 1. Introduction

Canine monocytotropic ehrlichiosis (CME) is an important infectious disease occurring in dogs. It is commonly transmitted by the brown dog tick, *Rhipicephalus sanguineus* [1,2]. The etiological agent of this disease is *Ehrlichia canis*, a small, gram-negative, obligatory intracellular bacterium in the family Anaplasmataceae [3]. The agent invades and propagates in monocytes and macrophages, which can then lead to a variety of hematological and clinical signs [4,5]. The acute phases of CME are characterized by high fever, depression, anorexia, lymphadenomegaly, and splenomegaly, as well as clinical signs that include dermal petechiae, ecchymosis, and epistaxis [6]. Thrombocytopenia is the most prominent hematological change that occurs in the acute phase [7]. The chronic phase is characterized by pancytopenia due to suppression or destruction of bone marrow [8]. The clinical signs in the chronic phase are similar to those of the acute phase but are associated with a greater degree of severity [6]. *E. canis* infections in dogs have been reported in North and South America, Europe, Africa, and Asia [9,10,11,12,13]. Additionally, there has been evidence of *E. canis* infections in humans in certain specific regions [14].

Vaccines for *E. canis* are needed; however, many obstacles have impeded their development including identification of ehrlichial antigens, an understanding of the relevant genetic and antigenic variabilities, and a lack of animal models that reflect the immune responses of the hosts [15]. Previous studies have shown that inactivated and attenuated vaccine candidates for CME were capable of provoking a humoral response, but only partial clinical protection was achieved in dogs challenged with the virulent strain [16,17]. The peptide vaccine is among a variety of modern vaccines that represent a potential strategy for the prevention and treatment of pathogenic diseases [18,19,20]. There are a number of noteworthy advantages associated with this vaccine relevant to its low cost, ease of synthesis, and inherent level of safety. All of these attributes are considered extremely attractive features [21]. In the case of *Ehrlichia* spp., recombinant P29 protein and P28-19 peptides have been developed and demonstrated an ability to protect against *E. muris* in mouse models [18,22]. Since there is no available vaccine against *E. canis*, evidence of the role of the humoral immune response in immunized dogs, compared with those that have been infected, is limited.

Ehrlichial antigenic proteins have been selected based on the degree of reactivity of the proteins with antibodies obtained from infected animals [23]. Major immunodominant *E. canis* antigens can be recognized early in the infection period as 19-, 37-, 75-, and 140-kDa proteins [24,25]. The 19-kDa protein has been identified as glycoprotein (GP19) and has revealed orthologs with variable-length PCR target proteins (VLPT) of *E. chaffeensis* [26]. *E. canis* GP19 consists of a serine/threonine/glutamate (STE)-rich patch at the amino-terminal that contains species-specific antibody epitopes that were strongly recognized by the serum collected from an *E. canis*-infected dog [26]. Additionally, GP19 was found to be one of the most conserved proteins (98.8–100% identities) among the geographically dispersed *E. canis* strains [27,28,29]. Due to the fact that a high degree of conservation and immunoreactive ability is attributed to *E. canis* GP19, this antigen has been recognized as an interesting target for diagnostic and vaccine development.

In this study, the GP19 epitope of *E. canis* was predicted. Subsequently, the GP19_4-43_ peptide was synthesized to produce hyperimmune serum to examine the capability of neutralization against *E. canis* infection in vitro. The canine macrophage-like cell line (DH82) that was isolated from a dog with histiocytic sarcoma was representative of permanent macrophage morphology and excellent phagocytic cells. Furthermore, this cell line has been reported for use in the successful culture and infection of *E. canis* [30,31]. 

In addition, cytokine mRNA expression levels involved with macrophage, humoral and cellular immune responses were evaluated. Different types of cytokines have been discovered including interferons (IFN), interleukins (IL), and the tumor necrosis factor (TNF) [32,33]. Expression of the cytokine subunit genes, particularly IFN-γ (*IFNG*), IL4 (*IL4*), IL-10 (*IL10*), IL-12 subunit beta (*IL12B*), IL-13 (*IL13*), and TNF (*TNF*), were investigated in this study to gain a greater understanding of the immunomodulatory action of the GP19_4-43_ antiserum against *E. canis* infection.

## 2. Materials and Methods

### 2.1. Construction of GP19_4-43_ Peptide

*E. canis* GP19 epitope sequences were determined based on protein conformation flexibility, hydrophilicity, and protein conformation. The prediction of the GP19 epitope containing the region was performed using Antibody Epitope Prediction-IEDB Analysis Resource (http://tools.immuneepitope.org/bcell/, accessed on 12 April 2020). A 40-amino-acid epitope sequence at the N-terminal region containing the STE-rich patch of *E. canis* GP19 was selected and then labeled as the GP19_4-43_ peptide. The 3D structure of the GP19_4-43_ peptide was predicted using Phyre2 protein modeling, prediction, and analysis [34] (Figure S1). The GP19_4-43_ peptide sequence was synthesized (Bio-Synthesis, Inc., Lewisville, TX, USA), resuspended in molecular biology grade water (0.8 mg/mL), and then kept at −20 °C until being used in the process going forward.

### 2.2. Production of Rabbit Antiserum against GP19_4-43_

Rabbit GP19_4-43_ antiserum was generated against the GP19_4-43_ peptide. New Zealand white rabbits were divided into 2 groups based on relevant immunogen formulations (*n* = 2 rabbits each group). The first group was comprised of two rabbits that were immunized with 100 µg of the GP19_4-43_ peptide/500 µL with an equal volume of Montanide (SEPPIC, Paris, France). The rabbits in the second group received only 500 µL of Montanide (control serum group). The immunized rabbits were intramuscularly administered 4 times at 2-week intervals. All rabbits were observed for clinical signs and behavioral changes that may have occurred at the pre- and post-immunization stages. These animal experiments were approved by the Animal Care and Use Committee (ACUC), approval No. S27/2560.

### 2.3. Evaluation of Rabbit Antibody against GP19_4-43_ Using ELISA

An indirect enzyme-linked immunosorbent assay (ELISA) was performed to monitor the specific rabbit sera IgG against the GP19_4-43_ peptide of *E. canis*. Briefly, 96-well immunoplates (Nunc-Immuno Plate MaxiSorp, Intermed, Roskilde, Denmark) were coated with the synthetic GP19_4-43_ peptide (1 ng/well) and then incubated overnight at 4 °C. The plates were washed three times with phosphate buffer saline (PBS) containing 0.05% Tween-20 (PBST) and then blocked with blocking buffer (5% skim milk in PBS) for 1 h. A volume of 100 μL of GP19_4-43_ immunized and control rabbit sera were added to each well; they were then incubated at 37 °C for 1 h. The plates were developed with secondary goat anti-rabbit IgG horseradish peroxidase (HRP) conjugated antibody (Dako Cytomation, Glostrup, Denmark) and incubated at 37 °C for 1 h. Subsequently, the 3,3′,5,5′-tetramethylbenzidine (TMB) substrate (SeraCare Life Sciences, Gaithersburg, MD, USA) was added to each well, and they were then incubated at room temperature for 15 min away from light. The reaction was stopped by adding 50 µL of 2 N H_2_SO_4_. Optical density (OD) was measured spectrophotometrically at 450 nm using an Accu Reader Microplate reader M965 (Metertech, Taipei, Taiwan R.O.C.). The cut-off value was determined following the formula employed in the previous study; cut-off value = mean negative control +3 standard deviations (SD) [35].

Rabbits were euthanized to collect blood samples one week after the last round of immunizations according to the AVMA Guidelines for the Euthanasia of Animals: 2020 Edition [36]. Blood samples were centrifuged to separate the sera and the pooled rabbit sera from each group were then stored at −20 °C until being used.

### 2.4. Canine Macrophage-Like Cell Lines and Culture Conditions

Canine macrophage-like cells (DH82, ATCC^®^ CRL-10389^TM^) were maintained in Dulbecco’s Modified Eagle Medium (DMEM; Gibco, Thermo Fisher Scientific, Waltham, MA, USA) that had been supplemented with 10% heat-inactivated fetal bovine serum (FBS; Gibco, Thermo Fisher Scientific, Waltham, MA, USA) in 25-cm^2^ tissue culture flasks at 37 °C and 5% CO_2_ in a humidified incubator. Five milliliters) in 25-cm^2^ tissue culture flasks at 37 °C and 5% CO_2_ in a humidified incubator. Five milliliters of cell-culture media were changed every 3–4 days. The cells were subcultured when about 90% confluence was reached; they were then observed under an inverted microscope (CK40; OLYMPUS, Olympus Optical, Tokyo, Japan). Cell detachment was performed using 1 milliliter of 0.25% Trypsin-EDTA, phenol red, 1X (Gibco, Thermo Fisher Scientific).

### 2.5. E. canis Culture and Stock Conditions

*E. canis* BF W053712X + 5 (PTA-5811™, ATCC^®^, Manassas, VA, USA) was used in this study and cultured in the 4th passage of DH82 according to the method employed in previous studies [4,37]. The monitoring of *E. canis* growth was performed at 1-week intervals by Giemsa staining via microscopic examination. Cells were propagated continuously until a level of 80% of infected cells was reached. Bacterial collection was performed manually by applying the previously described method [4]. Briefly, the approximate number of 5 × 10^6^ DH82 cells that were infected with *E. canis* were detached using 1 mL of 0.25% Trypsin-EDTA; they were then incubated for 15 min at 37 °C. An equal volume of DMEM was added to the flask and the cell suspension was passed 10 times through a bent 26 G needle to rupture the DH82 cells and release the intracellular bacteria mechanically. The suspension was centrifuged for 5 min at 1500× *g*. The supernatant containing cell-free bacteria was collected and the total volume was then adjusted to approximately 1 mL per 1 × 10^6^ cell-free *Ehrlichia*. The cell-free *Ehrlichia* was used in invasive detection and cytokine gene investigation in the next process. A total of 1 mL of the 1 × 10^6^ *E. canis*-infected DH82 cells were collected in 2-mL cryopreserved tubes with 5% dimethyl sulfoxide (DMSO) in DMEM and were then frozen at −80 °C until being used.

### 2.6. Infective Inhibition of Ehrlichia canis in DH82 Cell Line

#### 2.6.1. Infective Detection Using Light Microscopy

Detection of infective inhibition using light microscopy was modified from the method that had been previously described [38]. Briefly, DH82 cell viability and number were checked with 0.4% Trypan Blue. The approximate proportion of viable cells based on cytospin was found to be over 95%. The number of DH82 cells was adjusted to 1 × 10^5^ cells/mL and the cells were then deposited into 33 mm corning^®^ culture dishes (Corning Inc., Corning, NY, USA) and fixed with 22 × 22 mm coverslips. The cells were placed at the bottom of the wells and they were then incubated at 37 °C and 5% CO_2_ for 24 h to achieve cell adhesion. The dishes were then washed 3 times with 2 mL sterile 1 × PBS (pH 7.4) and used for *E. canis* invasive detection.

The treatment group was divided into 4 groups according to the different treatments. These different treatment groups included *E. canis*-infected DH82 cells (positive control group), *E. canis*-infected DH82 cells with control serum (untreated group), *E. canis*-infected DH82 cells with GP19_4-43_ antiserum (treated group), and uninfected DH82 cells (negative control group), respectively. A volume of 100 µL of 1 × 10^5^ cell-free *Ehrlichia* was added to 400 µL of the cell media (positive control group), 400 µL of control serum (untreated group), and GP19_4-43_ antiserum (treated group). The specimens were then incubated at 37 °C and 5% CO_2_ for 1 h. Then, the suspension was added to the monolayer of DH82 cells that were adjusted to 1 × 10^5^ cells in the dishes, and they were incubated at 37 °C and 5% CO_2_ for 24 h. Additionally, 1 × PBS solution was used as a negative control (uninfected DH82 cells). After 24 h, non-adherent bacteria were removed by washing the specimens twice with 2 mL of sterile 1 × PBS solution. After being washed, the coverslips were fixed in 4% formaldehyde solution and stained with Diff-Quik staining (Sigma-Aldrich, St. Louis, MO, USA). The experiments were performed in duplicate for each group. The coverslips were then observed under a light microscope.

#### 2.6.2. Infective Detection Using an Immunocytochemistry (ICC) Technique

The detection of *E. canis* infected cells was achieved following the ICC method applied in the previous study [39]. The number of DH82 cells were adjusted to 1 × 10^5^ cells per ml. Furthermore, 1 × 10^4^ DH82 cells in 100 µL cell culture media were cultured in duplicated 96-well plates for 24 h in a humidified incubator at 37 °C and 5% CO_2_ to allow for cell adhesion. The wells were then washed 3 times with 100 µL PBS/0.1% Tween-20 (PBST; pH 7.4) and used for invasive detection.

The treatment group was divided into 4 groups as has been mentioned above. For the positive control, and the untreated and treated groups, 100 µL of 5 × 10^4^ cell-free *Ehrlichia* was added to 200 µL of cell media, 200 µL of control serum, and GP19_4-43_ antiserum, respectively. The specimens were then incubated at 37 °C for 1 h. In the negative control group, PBS solution was used instead of *Ehrlichia*. Additionally, 100 µL (1 × 10^4^ cell-free *Ehrlichia*) of the suspension was added to the monolayer of DH82 cells in the media and incubated at 37 °C with 5% CO_2_ for 1 or 3 days. The media were then discarded, and non-adherent bacteria were removed by washing the bacteria three times with 100 µL of PBST. The cells were fixed with cold 4% paraformaldehyde for 15 min. Endogenous peroxidase was blocked using 3% H_2_O_2_ in 1 × PBS for 10–30 min. The cells were then blocked with 3% bovine serum albumin (BSA; AMRESCO, Solon, OH, USA) and washed three times with PBST. A solution comprised of 1:200 dilution of the primary antibody (rabbit GP19_4-43_ antiserum) was added to the plates and they were incubated at 4 °C overnight. The specimens were then washed once with PBST and incubated in a solution of 1:400 dilution of horseradish peroxidase (HRP)-conjugated goat anti-rabbit IgG (Sigma-Aldrich) for 1 h at RT. The plates were washed three times with PBST and then 75 µL of stable DAB was added (Invitrogen™, Carlsbad, CA, USA) for 2–5 min before being rinsed with tap water. The results were observed under an inverted microscope (400X, CK40; OLYMPUS, Shinjuku City, Tokyo, Japan) and infected cells were stained reddish-brown. A semi-quantitative scoring system for the stained cells was adopted from the system that had been previously described [40]. The average percentage of *E. canis* infected cells that were stained reddish-brown using the ICC technique was determined manually in 3 random fields and were then scored as follows; 0 = negative, 1 = 10% of the cells with positive staining, 2 = between 10–50% of the cells with positive staining, 3 = more than 50% of the cells with positive staining.

### 2.7. Cytokine Gene Expression Profiles

To investigate the effects of the GP19_4-43_ peptide on cytokine expression, a volume of 100 µL of 1 × 10^5^ cell-free *Ehrlichia* was added to 400 µL of the cell media (positive control group), control serum (untreated group), or GP19_4-43_ antiserum (treated group). The specimens were then incubated at 37 °C and 5% CO_2_ for 1 h. The suspension was added to the monolayer of 1 × 10^6^ DH82 cells in duplicate 24-well plates. In the negative control group, PBS solution was used instead of *Ehrlichia*. Plates were incubated at 37 °C, 5% CO_2_ for 1 or 3 days. After incubation, samples from each well were combined to achieve a cell density of 1 × 10^6^ cells. RNAlater^®^ solution was then added to the cells to preserve total RNA. RNA extraction was performed using a Total RNA Extraction Kit (RBCBioscience, Taipei, Taiwan) following the manufacturer’s instructions. RNA yields and concentrations were measured using a DU 730 nanoVette UV/Vis Spectrophotometer (Beckman Coulter, Brea, CA, USA). For cDNA synthesis, 2 μg of total RNA were used as a starter in 20 μL of a reaction mixture containing 2 μM Random Hexamer, 2.5 mM deoxynucleotide triphosphates (dNTPs), 5 × RT buffer, 40 U RNase inhibitor (Invitrogen™, Carlsbad, CA, USA), RNase-free water and 200 U Bioscript^TM^ reverse transcriptase (Bioline, Memphis, TN, USA). The cDNA synthetic reaction was carried out in a T100™ thermal cycler (Bio-Rad Laboratories, Hercules, CA, USA) according to the manufacturer’s instructions.

Approximately 50 ng of the cDNA samples were quantitatively analyzed for the mRNA transcription of gene coding that involved interferon-gamma (*IFNG*), interleukin 4 (*IL4*), interleukin 10 (*IL10*), interleukin 12B (*IL12B*), interleukin 13 (*IL13*), and tumor necrosis factor-alpha (*TNF*), while the glyceraldehyde 3-phosphate dehydrogenase (*GAPDH*) was used as a reference gene. Real-time RT-PCR was performed using a SensiFAST SYBR Hi-ROX Kit (Bioline, London, UK) following the protocol described by the manufacturer and by using CFX96 Touch™ Real-Time PCR (Bio-Rad, Hercules, CA, USA). Specific information regarding primer pairs used in this study is presented in Table S1. Sequences of the primers were designed by Primer3plus; primer synthesis was performed by Integrated DNA Technologies (IDT; Coralville, IA, USA). After gradient-PCR, the real-time RT-PCR cycling conditions were applied according to the initial denaturation at 94 °C for 5 min followed by 40 cycles at 94 °C for 30 s, 59 °C for 10 s and 72 °C for 20 s, ending with a final extension at 72 °C for 5 min for all genes. After completion of the assessment of the real-time RT-PCR cycles, dissociation curves were analyzed to confirm that the PCR product obtained by real-time RT-PCR included the correct components. The specificity of real-time RT-PCR on the specified genes was confirmed by 2% agarose gel electrophoresis. Analysis of relative gene expression was calculated from the Ct of the target gene and the reference gene (GAPDH). The expression levels (fold-difference) were reported using the 2^−ΔΔCt^ method [41].

### 2.8. Data Analysis

The data from all three experiments were combined and analyzed to identify any outliers using the robust regression and outlier removal (ROUT) method. The normality test was completed using the Shapiro-Wilk test prior to performing statistical analysis. Statistical analyses were performed using one-way ANOVA or multiple t-tests that employed GraphPad Prism 8.2.0 (GraphPad Software, Inc., San Diego, CA, USA). Results of the statistical analyses were considered significant in all experiments when *p* < 0.05. Multiple comparisons of each pair were made using the Holm–Sidak method. Results were reported as mean plus standard error (SE). The cytokine genes obtained from differential expression data were used to create a network of genes using CytoScape with a GeneMANIA plugin [42].

## 3. Results

### 3.1. Rabbit Antibody Response to GP19_4-43_ Peptide

**Figure 1:**
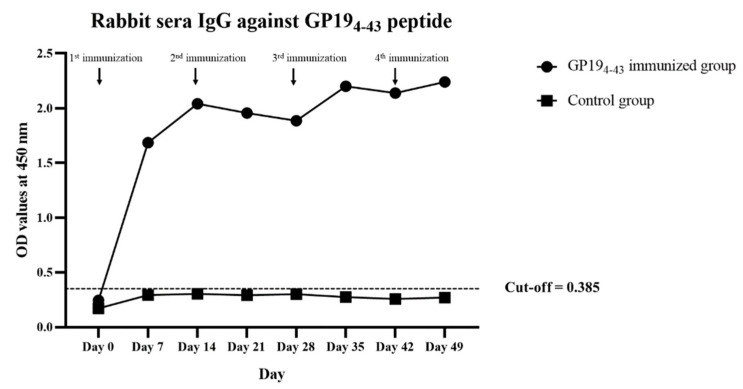
Antibody titer levels measured by indirect ELISA against GP19_4-43_ peptide non-immunization (control group) and immunization in rabbits throughout the experimental period.

All immunized and non-immunized rabbits displayed no clinical signs and behavioral changes at the pre- and post-immunization stages. The OD values of the immunized and control rabbit sera are shown in Figure 1. Following immunization of the rabbits, two peaks of antibody levels against the GP19_4-43_ peptide were detected 14 days after the first immunization and 7 days after the third immunization. The average OD values and SD of immunized rabbit sera weekly recorded at 0.240 ± 0.025, 1.661 ± 0.065, 2.037 ± 0.012, 1.960 ± 0.013, 1.885 ± 0.005, 2.202 ± 0.010, 1.982 ± 0.043 and 2.191 ± 0.022, respectively. The average OD values and SD of non-immunized control rabbit sera were 0.271 and 0.038, respectively. Thus, the cut-off point of the indirect ELISA detecting rabbit antibody against GP19_4-43_ of *E. canis* was 0.385.

### 3.2. Infective Inhibition of E. canis to DH82 Cell Line

#### 3.2.1. Positive *E. canis* Infection Detected by Light Microscopy

With the exception of the negative control group, *E. canis* cells infected with microorganisms within the membrane-bound compartments (morulae) were observed in the DH82 cytoplasm under light microscopy. This was attributed to a heavy degree of infection (1 × 10^5^ cell-free *Ehrlichia*) on day 1 of the post-infection period.

#### 3.2.2. *E. canis* Infective Inhibition Detected by ICC Technique

**Figure 2:**
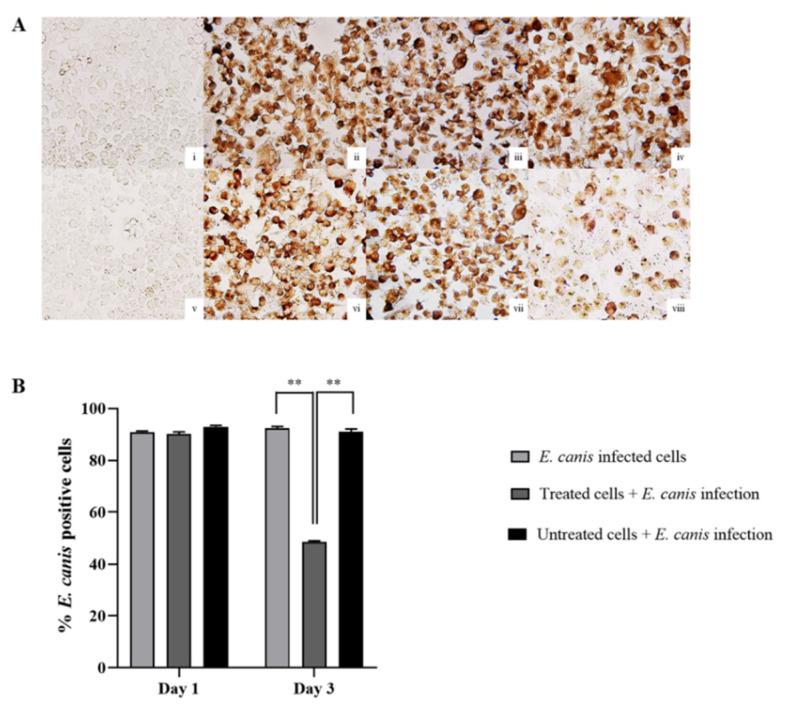
*E. canis* infective results in macrophage-like cells (DH82) using immunocytochemistry (ICC). (**A**) Representative micrographs of *E. canis* infective cells stained with DAB to indicate positive cells under an inverted microscope (400×, CK40; OLYMPUS): (**i**–**iv**) *E. canis* infective detection on day 1 post-infection in uninfected cells, *E. canis* infected cells, *E. canis*-infected cells with control serum (untreated group) and *E. canis*-infected DH82 cells with GP19_4-43_ antiserum (treated group), respectively; (**v**–**viii**) *E. canis* infective detection on day 3 post-infection in uninfected cells, *E. canis* infected cells, *E. canis*-infected cells with control serum (untreated group) and *E. canis*-infected DH82 cells with GP19_4-43_ antiserum (treated group), respectively. (**B**) Bar graphs represent the percentage of *E. canis*-infected cells on day 1 and day 3 post-infection in *E. canis* infected cells, *E. canis*-infected cells with control serum (untreated group) and *E. canis*-infected DH82 cells with GP19_4-43_ antiserum (treated group), respectively. Data are represented as mean ± SE, one-way ANOVA, ** *p* < 0.01.

With the exception of the negative control, infection of *E. canis* was graded with a score number of 3 (>50% positive cells) in all groups on day 1 of the post-infection period (Figure 2A). The percentages of *E. canis* infected cells in the positive control, and the treated and untreated groups, were 90.925 ± 1.178, 90.065 ± 2.772, and 92.918 ± 1.459, respectively (Figure 2B).

Notably, stimulated DH82 indicated different percentages of *E*. *canis* infected cells on the third day of the post-infection period (*p* < 0.01, Figure 2B). Furthermore, the treated group revealed lower percentages of positive staining cells with DAB staining (10–50% positive infected cells; score number 2. The percentages of *E. canis* infected cells in the positive control, treated and untreated groups were 92.291 ± 2.269, 48.573 ± 1.282, and 90.967 ± 3.132.

### 3.3. Relevance of GP19_4-43_ Antiserum on Cytokine Gene Expression

**Figure 3:**
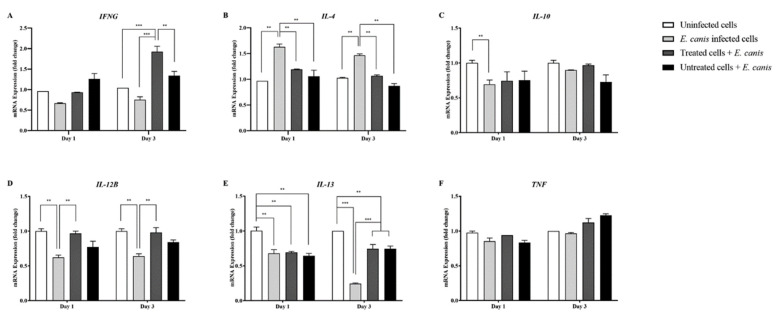
Real-time RT-PCR analyses of the canine macrophage-like cell line (DH82) mRNA expression involved in cytokine activity. Bar graphs showing relative (**A**) IFNG, (**B**) IL4, (**C**) IL10, (**D**) IL12B, (**E**) IL13, and (**F**) TNF mRNA expression levels after normalization to the GAPDH gene in uninfected cells, *E. canis* infected cells, *E. canis*-infected DH82 cells with GP19_4-43_ antiserum (treated group) and *E. canis*-infected cells with control serum (untreated group), respectively. Data is represented as mean ± SE (*n* = 4 each treatment), one-way ANOVA or multiple *t*-test, ** *p* < 0.01, *** *p* < 0.001.

Gene expression levels in the *E. canis*-infected group revealed overall down-regulation among cytokine genes IFNG, IL10, IL12B, and IL13 (Figure 3). In contrast, IL4 displayed a degree of upregulation (*p* < 0.01) when in the *E. canis*-infected group compared with all groups throughout the post-infection phase of the experiment (Figure 3B).

IFNG gene expression was maintained following *E. canis* infection in the presence of GP19_4-43_ antiserum with a degree of up-regulation of IFNG on day 3 (*p* < 0.001) when compared with uninfected and *E. canis*-infected group, likewise compared with *E. canis* infection in the control serum (*p* < 0.01) (Figure 3A). IL13 gene expression in the treated group revealed significantly downregulated levels of expression when compared to the uninfected group throughout the experiment (*p* < 0.01) (Figure 3E). The levels of gene expression of the other cytokine genes involved with the helper Th response (IL4, IL10, and IL12B) and the pro-inflammatory cytokine gene (TNF gene) in the treated group revealed no significant differences in the expression levels when compared to the uninfected group (Figure 3).

## 4. Discussion

Despite the importance of veterinary medicine and the possibility of zoonotic pathogens affecting humans, very few studies have been conducted on vaccine development against *E. canis* infection. GP19_4-43_ peptide serves as an immunogen in that it mimics the antigen structure, GP19 of *E. canis*, which can then elicit the antibody of infected dogs within 14 days. This model was constructed and used to produce rabbit GP19_4-43_ antiserum in the present study [21,43]. Additional immunizations (two-week intervals) were given to raise the concentration of the antibodies. After the 3rd round of immunizations, immunized rabbit antibody titers had risen to a peak within a week of the post-immunization period. Rabbit GP19_4-43_ antiserum containing a specific antibody was used to determine the effects of the GP19_4-43_ antiserum to macrophages against *E. canis* infection in the canine macrophage. The 4th passages of DH82 cells were representative of canine macrophage-like cells that could be used to control relevant cell characteristics, including cell infection and gene expression [30,44].

As was the case in previous studies, the passive transfer of serum containing *Ehrlichia*-specific immunoglobulin G (IgG) to *E. chaffeensis*-infected SCID mice pre- and post-infection displayed an effective degree of elimination of *Ehrlichia* [45,46]. Our present study reinforced the role of GP19_4-43_ antiserum against *E. canis* and showed the effects of elimination of *Ehrlichia* in canine macrophages on the third day of the post-infection period. Results of the current experiment suggest the possible importance of the following mechanism. Macrophages could be triggered by GP19_4-43_ antiserum with regard to the neutralizing activity against *E. canis* infection by a decrease in the number of positive infected cells by the third day of the post-infection period. Since this study focused on the early infection of *E. canis*, the effects of inhibition of *E. canis* infection of the macrophage-like cell line (DH82) in the presence of the antisera are still unknown in the context of long-term infection. Further studies on the neutralization role of the antibody against GP19 immunogens and *E. canis* infection, and between early and long-term infection, are needed to understand the effect of vaccine candidates against *E. canis* infection.

The expression of cytokine genes involved in macrophage-adaptive immune responses in DH82 cells was unraveled in this study. With the absence of GP19_4-43_ antiserum, the *E. canis* infected DH82 cells revealed significantly downregulated levels of expression of *IFNG* and *IL12B* genes that involved with macrophage-Th1 cells. These results were consistent with the findings of a previous study that had reported a low level of detection of IL-12p70 and IL-12p40 in mice spleen during virulent IOE lethal infection [47]. Contrastingly, the *IFNG* expression of *E. canis* infected cells in the presence of the GP19_4-43_ antiserum (treated group) displayed significantly up-regulated levels on day 3 when compared to all groups (Figure 3A). A decrease in *E. canis* infection was also observed on the third day of the post-treatment period; accordingly, the *IFNG* expression in the treated cell group could be related to the elimination of the intracellular microorganism, *E. canis*. Moreover, *IFNG* is the only cytokine gene investigated in this study that shows significantly different expression between the treated and untreated groups. The findings of the present study indicate the strong possibility that GP19_4-43_ antiserum was able to promote IFN-γ production in DH82 cells against *E. canis* infection. In addition, the outcomes of the present study support the use of IFN-γ as an indicator of protective immunity in animals immunized with *Ehrlichia* immunogens, as was evident from the findings of the previous studies [48,49].

The cytokine gene expression levels that were involved with macrophage-Th2 and B cells, including *IL4*, *IL10*, and *IL13* genes in DH82 cells, were also investigated. The results displayed no difference of these genes in uninfected cells, *E. canis* infected DH82 with and without GP19_4-43_ antiserum. Notable, the *E. canis* infected DH82 cells revealed upregulated levels of expression of *IL4* which reported a negative relationship with *IFNG* expression. With regard to vaccine development against *Ehrlichia* spp., the previous study revealed that the killed vaccine of *E. ruminantium* elicited both CD4+ and CD8+ subsets to produce IFN-γ in the absence of IL4, indicating a type 1 response [50]. However, the relation of these two genes has not been reported in canine macrophages. Investigation of the macrophage-adaptive immune response in animals immunized with the GP19_4-43_ peptide is further needed for a clearer understanding of the protective ability.

The overproduction of IL-10 and TNF-α was reported to be associated with toxic shock-like syndrome and mortality in animal models of fetal monocytic ehrlichiosis, according to the findings of previous studies [47,51,52,53]. In the present study, there were no significant differences in *IL10* and *TNF* gene expression levels among *E. canis* infected cells (positive, treated, and the untreated group). Further studies are recommended on the expression of the anti-inflammatory and pro-inflammatory cytokine genes in various cells or animal models over a longer period of time to reach a definitive conclusion on the effects of the antiserum on these genes.

**Figure 4:**
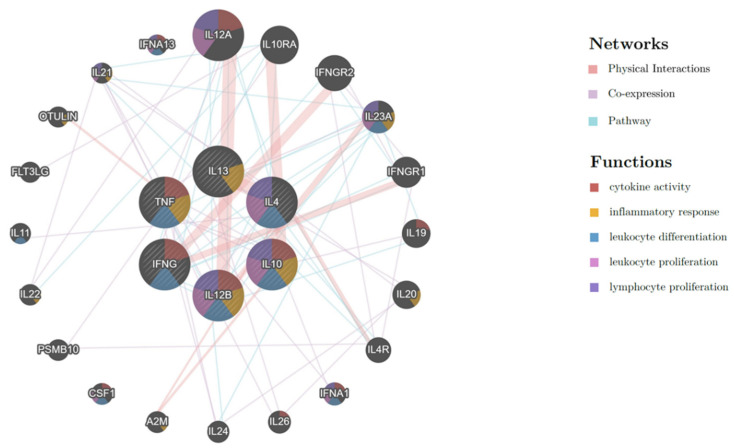
GeneMANIA Cytoscape interaction network of known physical interactions, gene co-expression, and cytokine pathway with query genes (IFNG, IL4, IL10, IL12B, IL13, and TNF) that were expressed in this study were found to be involved with the function of cytokine activity (brown nodes), inflammatory response (yellow nodes), leukocyte differentiation (blue nodes), leukocyte proliferation (lilac nodes) and lymphocyte proliferation (violet nodes). Additional genes are predicted to be involved in the network (gray nodes).

Data on the gene expression levels for both up- and down-regulation trends were used to analyze the network of relationships using GeneMANIA plug-ins [54]. The results revealed evidence of a relationship among query genes in this study (*IFNG*, *IL4*, *IL10*, *IL12B*, *IL13,* and *TNF*), as well as among the genes in the public dataset of functional association networks. Predictions of known gene co-expression and physical interactions in the cytokine activity pathway with query genes (brown nodes), leukocyte differentiation (blue nodes), leukocyte proliferation (lilac nodes), lymphocyte proliferation (violet nodes), and inflammatory responses (yellow nodes) were defined by the presence of additional genes that are related to the individual query genes (Figure 4). In addition, some genes were indirectly present in the network, namely *CSF1*, *IL10RA*, *DGKA*, *IFNGR1*, *IFNGR2*, *IL21R*, *IL24*, *IL4R*, and *FLT3LG*. Our analyses of the cytokine networks suggest the possible linkages of the cytokine genes identified in this study with additional cytokines involved in the *E. canis* infection responses with the collaboration of other macrophages and other immune cells. Therefore, cytokine detection in different immune cells, including macrophages, CD4+ and CD8+ T cells, observed in animal models would be needed to better understand the role of the GP19_4-43_ peptide as an immunogen in the development of a vaccine against *E. canis*.

## 5. Conclusions

Synthetic GP19_4-43_ peptide was used as an immunogen to produce rabbit GP19_4-43_ hyperimmune serum in the present study. The GP19_4-43_ antiserum was examined for *E. canis* infective inhibition in macrophage-like cells (DH82). A decrease in ehrlichial infection was observed in the GP19_4-43_ antiserum treated group when compared with the positive control and the untreated group on the third day of the post-infection period, as was observed through the use of the ICC method. Cytokine genes involved in macrophage-adaptive immune responses were also investigated. The present findings highlight the expression level of *IFNG* displaying marked up-regulation in the treated group. These findings can fill the existing gaps in terms of the recorded knowledge of immune response against *E. canis* infection in both treated and untreated macrophages with GP19_4-43_ antiserum containing the specific antibody. This study also indicates potential directions for future studies on the genes involved in phagocyte-cell mediated immune response. Notably, the relative macrophage, humoral and cellular immune response in both in vitro and in vivo studies will be needed for further verification.

## Data Availability

All data in this study have been included in the manuscript.

## Supplementary Materials

The following are available online at https://www.mdpi.com/article/10.3390/ani11082310/s1, Figure S1: Amino acid sequence of *E. canis* GP19_4-43_ peptide, Table S1: PCR and oligonucleotide sequences of primers targeting cytokine genes used in this study.
