# Supplementary material for: Effect of GP19 Peptide Hyperimmune Antiserum on Activated Macrophage during Ehrlichia canis Infection in Canine Macrophage-like Cells
Source: Animals (Basel). 2021 Aug 5;11(8):2310. doi: 10.3390/ani11082310 (PMC8388390; doi:10.3390/ani11082310)
Supplement: Supplementary file 1 [file animals-11-02310-s001.zip › Nambooppha_Supplementary file_R1.pdf]

Supplementary figures and table

Figure S1. Amino acid sequence of *E. canis* GP19$_{4\text{-}43}$ peptide corresponding to the underlined predicted epitope sequence was synthesized. (A) The peptide corresponding to the bold underlined sequence was chosen for synthesis to induce the polyclonal antibody in rabbits (GP19$_{4\text{-}43}$ antiserum). (B) epitope region prediction using Antibody Epitope Prediction-IEDB Analysis Resource (http://tools.immuneepitope.org/bcell/). (C) The model of GP19$_{4\text{-}43}$ Predicted 3D structure using Phyre2 protein modeling, prediction and analysis (Kelley et al., 2015).

GP19$_{4\text{-}43}$

A

```
1    MLHVQNHVDQ  HTNHIEHDDY  HFTGPTSFEV  NLSEEEKMEL  QEVSSIDSVG  CEDCDPNCRY
61   PLELVECQRI  EERPVCNAGL  ESLTVDAYQL  GLLLGGFLSA  MNYISYSYPC  YYYDCCDRNY
121  YDCCHKNACY  YNCCDCA
```

B

C

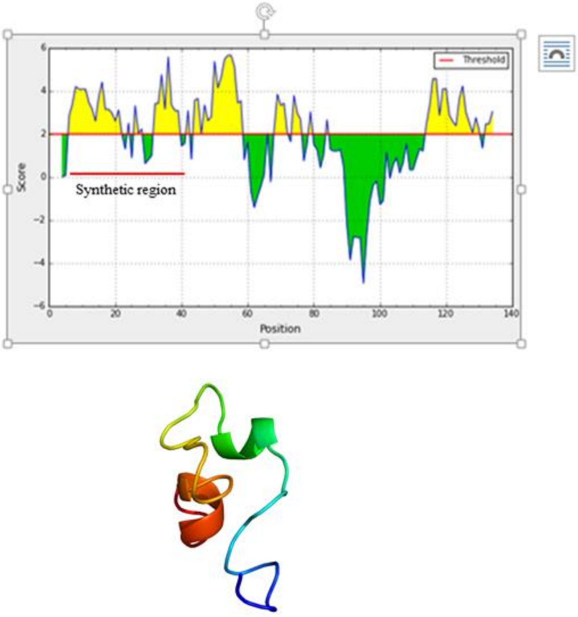

Table S1. PCR and oligonucleotide sequences of primers targeting cytokine genes used in this study.

Primer name	Oligonucleotide sequence (5'-3')	Target gene[A]	Product size
**dIFNG-F**	5'-CGG TGG GTC TCT TTT CGT AG-3'	*IFNG*	198
**dIFNG-R**	5'-TCC CTC TTA CTG GTG CTG CT-3'		
**dIL4-F**	5'-CTC ACC TCC CAA CTG ATT CC-3'	*IL4*	185
**dIL4-R**	5'-CTT GTG TTC TTT GGG GCA GT-3'		
**dIL10-F**	5'-CCT GGG AGA GAA GCT CAA GA-3'	*IL10*	221
**dIL10-R**	5'-TGT TCT CCA GCA CGT TTC AG-3'		
**dIL12B-F**	5'-CCG CTC ACT CCT GTT GAT TC-3'	*IL12B*	229
**dIL12B-R**	5'-TGT CAC TGC TCC ACA TGT CA-3'		
**dIL13-F**	5'-ATC ACC CAG AAT CAG GCA TC-3'	*IL13*	195
**dIL13-R**	5'-GCT GCG TTC ACT GGA AAT CT-3'		
**dTNFA-F**	5'-ACC ACA CTC TTC TGC CTG CT-3'	*TNF*	237
**dTNFG-R**	5'-CTG GTT GTC TGT CAG CTC CA-3'		
**dGAPDH-F**	5'-ATC ACT GCC ACC CAG AAG AC-3'	*GAPDH*	202
**dGAPDH-R**	5'-GGC AGG TCA GAT CCA CAA CT-3'		

[A] The abbreviation of target genes regarding *Canis lupus familiaris*. *IFNG*; interferon-gamma, *IL4*; interleukin 4, *IL10*; interleukin 10, *IL12B*; interleukin 12B (subunit p40), *IL13*; interleukin 13, *TNF*; tumor necrosis factor, *GAPDH*; Glyceraldehyde 3-phosphate dehydrogenase.
